# Mitral Valve Surgery for Mitral Regurgitation Results in Reduced Left Ventricular Ejection Fraction in Barlow’s Disease as Compared with Fibro-Elastic Deficiency

**DOI:** 10.3390/jcdd11030071

**Published:** 2024-02-21

**Authors:** Lobke L. Pype, Philippe B. Bertrand, Philippe Debonnaire, Sebastiaan Dhont, Boukje Hoekman, Bernard P. Paelinck, Dina De Bock, Hein Heidbuchel, Emeline M. Van Craenenbroeck, Caroline M. Van De Heyning

**Affiliations:** 1 Department of Cardiology, Antwerp University Hospital, 2650 Edegem, Belgium; lobke.pype@uza.be (L.L.P.);; 2 GENCOR Research Group, University of Antwerp, 2000 Antwerp, Belgium; 3 Department of Cardiology, Hospital Oost-Limburg, 3600 Genk, Belgium; 4 Faculty of Medicine and Life Sciences, Hasselt University, 3500 Hasselt, Belgium; 5 Department of Cardiology, Sint-Jan Hospital Bruges, 8000 Bruges, Belgium; 6 Department of Cardiac Surgery, Antwerp University Hospital, 2650 Edegem, Belgium

**Keywords:** mitral valve prolapse, mitral regurgitation, mitral valve annuloplasty, mitral valve replacement, left ventricular remodeling

## Abstract

Surgical correction of severe mitral regurgitation (MR) can reverse left ventricular (LV) remodeling in patients with mitral valve prolapse (MVP). However, whether this process is similar to the case in Barlow’s Disease (BD) and Fibro-elastic Deficiency (FED) is currently unknown. The aim of this study is to evaluate post-operative LV reverse remodeling and function in patients with BD versus FED. In this study, 100 MVP patients (BD = 37 and FED = 63) with severe MR who underwent mitral valve surgery at three Belgian centers were retrospectively included. Transthoracic echocardiography was used to assess MR severity, LV volumes and function before surgery and 6 months thereafter. Baseline MR severity, LV ejection fraction (LVEF), indexed LV end-diastolic (LVEDVi) and end-systolic volumes (LVESVi) were not different between the groups. After a median follow-up of 278 days, there was a similar decrease in LVEDVi, but a trend towards a smaller decrease in LVESVi in BD compared to FED (−3.0 ± 11.2 mL/m^2^ vs. −5.3 ± 9.0 mL/m^2^; *p* = 0.154). This resulted in a significantly larger decrease in LVEF in BD (−8.3 ± 9.6%) versus FED (−3.9 ± 6.9%) after adjusting for baseline LVEF (*p* < 0.001) and type of surgical intervention (*p* = 0.01). These findings suggest that LV (reverse) remodeling in BD could be affected by other mechanisms beyond volume overload, potentially involving concomitant cardiomyopathy.

## 1. Introduction

Mitral valve prolapse (MVP) is the most common cause of primary mitral regurgitation (MR) and has a prevalence of 2–3% in the general population [1]. While the disease course of MVP appears to be benign in some patients, chronic MR can cause substantial volume and pressure overload, subsequently leading to severe remodeling with left ventricular (LV) dilatation and/or dysfunction [2,3]. MVP can be subcategorized as primarily related to Fibro-elastic Deficiency (FED) or to Barlow’s Disease (BD), the two ends of the MVP spectrum. Patients with FED typically present with isolated leaflet or segment prolapse due to degenerative processes, whereas BD is characterized by bileaflet prolapse with thick and myxomatous valvular infiltration [4,5]. Interestingly, LV dilation in FED is thought to result entirely from volume overload in the presence of severe MR. However, significant LV dilation has been observed in BD even in the absence of MR, potentially involving an underlying cardiomyopathy in certain patients with BD [6,7,8]. The evolution of LV dilatation after correction of MR could further underscore this hypothesis.

In the presence of severe MR and associated symptoms or markers of LV dilation and/or dysfunction, mitral valve surgery is indicated to eliminate volume overload and reverse the process of LV remodeling [9,10]. Nevertheless, it remains unknown whether LV reverse remodeling following mitral valve surgery is similar in both MVP subtypes, especially since other factors could affect the process of LV (reverse) remodeling beyond volume overload in patients with BD [3,7]. Therefore, this study aims to evaluate the impact of the MVP subtype on LV reverse remodeling after mitral valve surgery.

## 2. Materials and Methods

**Figure 1:**
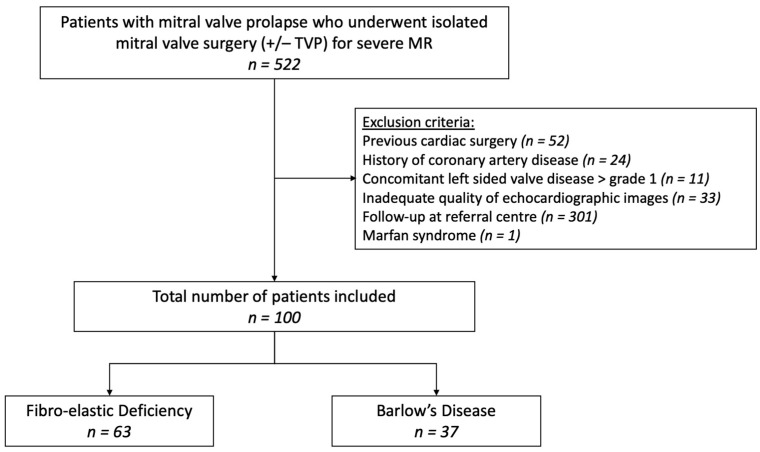
Flowchart patient selection. MR, mitral regurgitation; TVP, tricuspid valve annuloplasty.

We retrospectively included all consecutive patients with mitral valve prolapse (MVP) who underwent isolated mitral valve surgery (+/− tricuspid valve annuloplasty) in 3 Belgian referral centers: University Hospital Antwerp (from January 2013 to October 2022), Hospital Oost-Limburg (from January 2017 to October 2022) and Sint-Jan Hospital Bruges (from January 2019 to December 2021). Approval of the central ethical committee (UZA) was obtained. All eligible patients had to (1) have (moderate-to) severe primary MR, (2) be age 18 years or older, and (3) have comprehensive echocardiography at baseline and after a minimum of 6 months post-operative follow-up. Subjects were excluded if they presented with any of the following: (1) history of cardiac surgery, (2) coronary artery disease, (3) significant concomitant left-sided valve disease (≥moderate aortic stenosis/insufficiency and/or ≥moderate mitral stenosis), (4) syndromic MVP (e.g., Marfan’s syndrome), (5) congenital heart disease, (6) inadequate quality of echocardiographic images;, and (7) follow-up at a referral center (Figure 1).

Clinical characteristics were collected from the electronic patient files. Patients were classified as BD or FED according to the intra-operative diagnosis of myxomatous mitral valve (=BD) or overall normal valvular tissue (=FED). Ventricular arrhythmias were defined as a reported history of ventricular tachycardia (minimum of 3 consecutive beats) or ventricular fibrillation; screening with Holter was not performed. The baseline preoperative echocardiography and first follow-up echocardiography after a minimum of 6 months were analyzed. LV end-diastolic and end-systolic volumes and ejection fractions (LVEFs) were calculated using the modified Simpson’s method. All ventricular and atrial volumes were indexed to body surface area (LVEDVi and LVESVi, respectively). The change in volumes and function was expressed as ΔLVEDVi (LVEDVi_follow-up_ − LVEDVi_baseline_), ΔLVESVi (LVESVi_follow-up_ − LVESVi_baseline_) and ΔLVEF (LVEF_follow-up_ − LVEF_baseline_). In addition, the relative change in LVEF was calculated as ΔLVEF/LVEF_baseline_.

A multi-integrative approach was used to quantify MR severity. Regurgitant volume (Rvol) was calculated using the proximal isovelocity surface area (PISA) method. MR severity was classified as no/trace (grade 0), mild (grade 1), moderate (grade 2), moderate to severe (grade 3) or severe (grade 4) [11]. The prolapse volume, defined as the end-systolic volume between the mitral annular plane and mitral valve leaflets, was calculated from the apical 4-chamber and 2-chamber views, as described previously [12]. Systolic pulmonary artery pressures were calculated from the maximal tricuspid regurgitant jet velocity and an estimate of the right atrial pressure based on inferior caval vein dimension and collapsibility. All echocardiographic images were analyzed by a single observer per center.

Continuous variables are expressed as mean ± standard deviation for normally distributed variables or median with interquartile range (IQR) for parameters with a non-normal distribution. Categorical variables are expressed as numbers and percentages and were compared using a Chi-square test or Fisher’s exact test. Baseline and follow-up LV volumes and LVEF (in the 2 subgroups separately) were compared with a paired Student’s *t*-test or nonparametric alternative (Wilcoxon signed-rank test). Comparisons between the 2 MVP subtypes were performed with a Student’s *t*-test or nonparametric alternative (Mann–Whitney U test). Follow-up echocardiographic variables were corrected for their respective baseline variables using a simple linear regression analysis. Univariable and multivariable linear regression analyses were conducted to identify the determinants of post-operative change in LVEFs. Only variables with a *p*-value of ≤0.05 on univariate analysis were included in the multivariable linear regression analysis. Additional multicollinearity statistics were gathered (variance inflation factors and tolerance) to exclude a significant correlation between the independent determinants in the multivariable regression analysis. Statistical analyses were performed using SPSS version 28.0 (SPSS Inc., Chicago, IL, USA). A *p*-value of ≤0.05 was considered statistically significant.

## 3. Results

### 3.1. Baseline Characteristics

A total of 100 patients were included, 37 with BD and 63 with FED (Figure 1). The baseline clinical characteristics are shown in Table 1. Patients with FED tended to be older compared to BD and had a marked male predominance and a higher body mass index; however, body surface area was not significantly different. The presence of symptoms, cardiovascular risk factors, family history of valvular heart disease and atrial fibrillation was similar in both groups. Furthermore, there was no significant difference regarding the prevalence of ventricular arrhythmias between BD and FED.

### 3.2. Baseline Echocardiography

Baseline echocardiographic characteristics are presented in Table 2. At baseline, BSA-indexed LV end-diastolic (LVEDVi) and end-systolic volume (LVESVi) were similar between BD and FED. In addition, preoperative MR severity was not significantly different between both groups. Patients with BD presented with a larger prolapse volume and a larger mitral annular diameter compared to patients with FED, while chordal rupture was more prevalent in FED. Furthermore, the left atrial volume index was similar in both groups, and there was no significant difference in systolic pulmonary artery pressures between BD and FED.

### 3.3. Surgical Intervention

Most patients underwent mitral valve repair (76%) using one or more of the following techniques: mitral valve annuloplasty, Neochord and/or triangular/quadrangular resection (Table 3). Mitral valve replacement (MVR) was performed in selected patients when valve repair was not feasible, mainly in BD (46% vs. 11%; *p* < 0.001). Most patients undergoing MVR received a mechanical mitral valve prosthesis (58%). Additional tricuspid valve annuloplasty was performed in 9% of patients, with no difference between BD and FED. In selected patients with a history of atrial fibrillation, surgical pulmonary vein isolation (6%) and/or left atrial appendage occlusion (21%) was additionally performed with no difference among the MVP groups. At discharge, significant residual MR (grade ≥ 2) was observed in four FED patients.

### 3.4. Follow-Up Echocardiography

**Figure 2:**
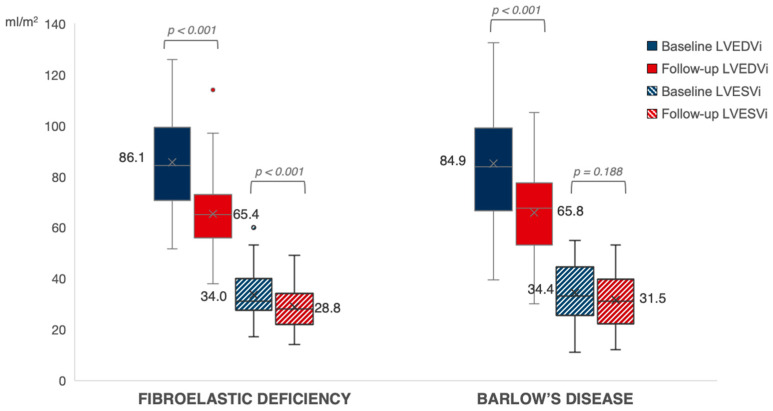
Evolution of left ventricular volumes in FED vs. BD. The evolution of LV end-diastolic and end-systolic volumes at baseline (in blue) towards follow-up (in red) in patients with FED versus BD. Expressed values are mean (shown as “x”). LVEDVi, left ventricular end-diastolic volume index; LVESVi, left ventricular end-systolic volume index.

**Figure 3:**
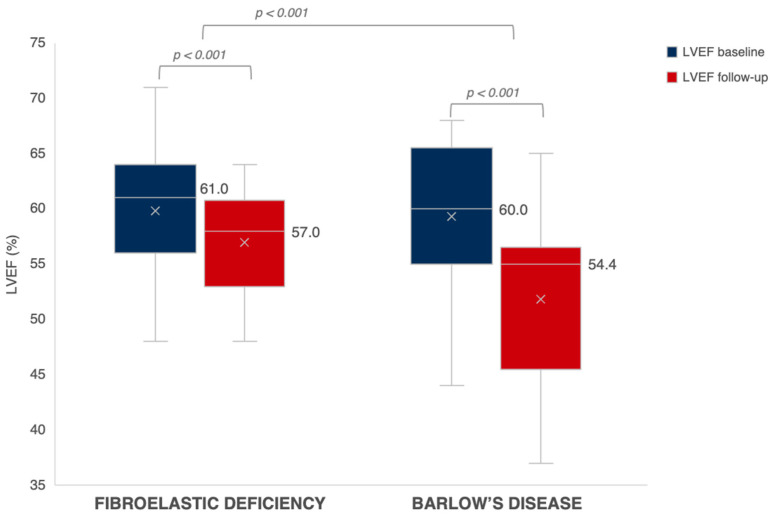
Evolution of LVEF in FED vs. BD. The evolution of LVEF at baseline (in blue) towards follow-up (in red) in patients with FED versus BD. Expressed values are median. The mean is shown as “x”. LVEF, left ventricular ejection fraction.

Follow-up echocardiographic characteristics are presented in Table 4. After a median follow-up period of 278 days in BD and 288 days in FED (*p* = 0.753), there was no difference between both groups regarding the presence of residual MR ≥ grade 2. We observed a significant decrease in LVEDVi from baseline to follow-up in both BD and FED (*p* < 0.001). While patients with FED experienced a significant post-operative decrease in LVESVi as well (*p* < 0.001), there was only a limited decrease in the less load-dependent LVESVi in patients with BD from baseline to follow-up (*p* = 0.188) *(*Figure 2). At follow-up, we observed a larger decrease in LVEF in BD as compared to FED after correction for baseline LVEF (*p* < 0.001) (Figure 3). A LVEF of < 50% was present in 28% of patients with BD compared to 12% in FED (*p* = 0.050); however, this difference did not remain statistically significant after correction for baseline LVEF (*p* = 0.060). Of note, we observed a limited decrease in LVESVi and significantly lower post-operative LVEF in patients with mitral valve replacement compared to mitral valve repair (respectively 0.2 ± 12.4 mL/m^2^ vs. −5.8 ± 8.6 mL/m^2^, *p* = 0.025 and −10.1 ± 9.2% vs. −4.2 ± 7.5%, *p* = 0.005). Additional subgroup analysis was performed excluding patients who underwent valve replacement without preservation of the subvalvular apparatus (*n* = 90), which showed no significant difference between LVEF decrease at follow-up between mitral valve replacement (−8.1 ± 10.1%) versus mitral valve repair (−4.2 ± 7.5%) (*p* = 0.091). Furthermore, this subgroup analysis presented with similar results regarding the evolution of post-operative LVEF in BD compared to FED (−6.8 ± 9.9% vs. −3.9 ± 6.9%; *p* = 0.006). Of note, subgroup analysis of only patients that underwent mitral valve repair (*n* = 76) showed a clear trend but not a statistically significant difference between LVEF decrease in BD versus FED (−5.7 ± 8.5% vs. −3.7 ± 7.1%, *p* = 0.059) (Supplementary Table S1).

Table 5 shows the univariable linear regression analyses used to identify the predictors of post-operative LVEF decrease (ΔLVEF). Based on these univariable regression analyses, baseline LVEF, baseline LVESVi, MVR and BD were significantly associated with a greater decrease in LVEF after follow-up. Multivariable linear regression analysis showed that only baseline LVEF and MVP subtype remained a significant determinant of post-op change in LVEF (Table 6). Additional multicollinearity statistics verified that the predictors of BD and MVR were not closely correlated with one another. Thus, the effect of the MVP subtype on LVEF cannot be explained by the higher rate of MVR in BD. Of note, ΔLVEF was independent of age, sex, comorbidities, medical therapy and symptoms.

## 4. Discussion

The present study investigated reverse remodeling in MVP patients undergoing mitral valve surgery for severe MR. The key findings are that (1) the BD subtype is independently associated with a reduced LVEF recovery after intervention, even after correction for baseline LVEF and type of surgical intervention, and (2) while baseline LVESVi and MVR were associated with ΔLVEF in the univariable regression analysis, they were not independently associated with post-operative change in LVEF.

In 2019, Le Tourneau et al. described the phased process of LV reverse remodeling after mitral valve surgery in primary MR. In short, the post-operative reduction in MR regurgitant volume results in an abrupt decrease in LV preload. Since LVEDV is known to be largely load-dependent, the early reverse remodeling phase is characterized by a decline in LVEDV, while LVESV does not change yet, resulting in a decreased stroke volume (LVEDV − LVESV). Accordingly, LVEF (stroke volume/LVEDV) will decline as well. After several months, LV end-systolic volume usually decreases towards normal values with recovery of LVEF [13]. The observed LVESVi and LVEF at >6 month follow-up in FED patients in our cohort is in line with this late reverse remodeling phase. In contrast, the BD patients in our study showed a trend towards less favorable reverse remodeling with higher LVESVi and lower LVEF at follow-up.

Furthermore, our study confirms the recent findings of Althunayyan et al. that LV dysfunction after mitral valve surgery is not uncommon in patients with MVP [14]. In our study, clinically significant LV dysfunction with LVEF < 50% at follow-up [15] was more prevalent in patients with BD compared to FED, but this difference did not remain significant after correction for baseline LVEF.

Moreover, patients with BD had a higher rate of MVR compared to FED, which has previously been associated with a lower post-op LVEF compared to MV repair [16], yet MVP subtype remained a significant determinant for change in LVEF even after correction for surgical intervention. In fact, effective LV contraction is partially supported by the dynamic relationship between the mitral valve annulus (including papillary muscles and chorda tendineae) and the LV free wall. Therefore, disrupting this annulo-ventricular continuity due to the (partial) removal of the subvalvular apparatus during MV replacement is hypothesized to result in less favorable post-operative LV geometry and function compared to MV repair. However, MVR with the preservation of the papillary muscles and/or chordae tendineae is associated with similar post-operative LV reverse remodeling compared to MV repair, as was recently observed by Craven et al. [17]. Their findings are in line with our data and subgroup analysis excluding patients without MV subvalvular apparatus preservation, which confirmed that BD was associated with a significantly larger post-operative decrease in LVEF compared to FED.

Therefore, the question arises whether the previously observed effect of MVR on post-operative LVEF could be partially explained by the higher prevalence of BD together with regular resection of the subvalvular apparatus in prior surgical cohorts, but thus far, this remains only a hypothesis.

What explains the difference in post-operative LV remodeling in BD versus FED remains to be elucidated; however, a number of observations deserve attention. First, BD is generally associated with longstanding LV volume overload due to chronic MR compared to the more (sub)acute MR following chordal rupture in FED. Therefore, patients with BD may present with already more structural LV adaptation in the preoperative phase compared to FED. Yet, we need to recognize that a difference in the time course of MR cannot be the complete explanation since significant LV remodeling was observed by Malev et al. in a very young cohort of BD patients without longstanding volume overload. Second, several studies have demonstrated that BD can be associated with severe LV dilatation and dysfunction as well as myocardial fibrosis, even in the absence of significant MR [6,7,8,18]. Third, BD has been associated with an underlying genetic substrate [19,20] and ventricular arrhythmias, including frequent premature ventricular contractions [21], which could contribute to dilated cardiomyopathy. Finally, El-Tallawi et al. suggested that the disproportionate LV dilatation in BD could be explained by a larger total volume load, defined as the sum of the MR volume and prolapse volume [22,23]. Due to annular dilatation and bileaflet prolapse in BD, these patients can present with a significant prolapse volume, but this is a rather unlikely explanation of the limited post-op reverse remodeling in BD since the prolapse volume is also corrected by mitral valve surgery.

Consequently, we hypothesize that other factors beyond volume overload affect the process of LV (reverse) remodeling in patients with BD. The presence of an underlying genetic and/or ectopy-induced cardiomyopathy could potentially elucidate why the successful elimination of volume overload through mitral valve surgery cannot completely reverse LV remodeling in some patients with BD. Furthermore, we could even speculate that a theoretically non-severe MR volume may be hemodynamically significant in a patient with BD due to their underlying myocardial substrate, which potentially suggests a benefit for early intervention in this patient group. Yet, this is currently no more than a hypothesis that requires further investigation.

We tried to overcome the inherent limitations of the retrospective study design by screening consecutive cases in three centers. Despite the multicenter aspect of the study, the sample size is rather small. The main reason for drop out was if patients received their echocardiographic follow-up with the referring cardiologist. Therefore, we expect no significant effect of patient selection on the differences between MVP subtypes. In addition, the ratio of included FED to BD patients (2:1) is similar to what would be expected in the general population. In addition, the rate of mitral valve replacement in this study (especially for BD patients) was higher than in previously published cohorts. Therefore, outcomes might be different in centers with higher repair rates for BD. Importantly, we only had access to the available standard 2D echocardiographic images that were obtained in clinical routine, while 3D echocardiography and cardiac magnetic resonance imaging are known to be superior in the assessment of LV volumes [24].

## 5. Conclusions

While patients with BD and FED showed similar MR severity, LV volumes and LVEF at baseline, the post-operative recovery of LVEF was significantly smaller in patients with BD, even after correction for baseline values and surgical intervention. These findings suggest that LV (reverse) remodeling in BD could be affected by other mechanisms beyond volume overload, including concomitant cardiomyopathy.

## Figures and Tables

**Table 1:** Baseline clinical characteristics.

	All Patients (*n* = 100)	BD (*n* = 37)	FED (*n* = 63)	*p*-Value (BD vs. FED)
Male (%)	71 (71.0)	21 (56.8)	50 (79.4)	**0.016**
Age (years old)	60 ± 12	57 ± 13	62 ± 11	0.066
BMI (kg/m^2^)	25.2 ± 3.9	23.6 ± 3.0	26.2 ± 4.1	**<0.001**
BSA (m^2^)	1.92 ± 0.24	1.87 ± 0.24	1.96 ± 0.23	0.088
Smoking (%)	32 (32.0)	10 (27.8)	22 (34.9)	0.465
Arterial hypertension (%)	46 (46.0)	14 (37.8)	32 (50.8)	0.209
Hypercholesterolemia (%)	33 (33.0)	12 (32.4)	21 (33.3)	0.926
Diabetes (%)	2 (2.0)	0 (0)	2 (3.2)	0.274
Chronic kidney disease (%)	8 (8.0)	2 (5.4)	6 (9.5)	0.464
Familial history of CV disease (%)	44 (44.0)	16 (48.5)	28 (49.1)	0.953
Familial history of valve disease (%)	10 (10.0)	5 (16.7)	5 (8.9)	0.286
Heart failure (%)	4 (4)	2 (5.4)	2 (3.2)	0.583
Atrial fibrillation (%)	30 (30.0)	12 (32.4)	18 (28.6)	0.684
**Medication**				
Beta-blocker (%)	41 (41)	11 (30.6)	30 (47.6)	0.097
ACE-I or ARB (%)	44 (44)	14 (38.7)	30 (47.6)	0.400
Anticoagulation (%)	18 (18.0)	6 (16.7)	12 (19.0)	0.768
MRA (%)	11 (11.0)	2 (5.6)	9 (14.3)	0.184
Non potassium sparing diuretics (%)	20 (20.0)	4 (11.1)	15 (23.8)	0.123
**Symptoms**				
Symptomatic (%)	78 (78.0)	29 (82.9)	49 (81.7)	0.884
Dyspnea (%)				0.335
NYHA class I	28 (28.0)	11 (31.4)	17 (28.3)	
NYHA class II	40 (40.0)	12 (34.3)	28 (46.7)	
NYHA class III	19 (19.0)	10 (28.6)	9 (15.0)	
NYHA class IV	8 (8.0)	2 (5.7)	6 (10.0)	
Chest pain (%)	25 (25.0)	9 (25.7)	16 (26.7)	0.919
Palpitations (%)	40 (40.0)	19 (54.3)	21 (35.0)	0.066
Syncope (%)	6 (6.0)	3 (8.6)	3 (5.0)	0.490

Values are mean ± standard deviation, *n* (%) or median (IQR). *p*-values ≤ 0.05 are expressed in bold. ACE-I, angiotensin-converting enzyme inhibitor; ARB, angiotensin receptor blocker; BMI, body mass index; BSA, body surface area; COPD, chronic obstructive pulmonary disease; MRA, mineralocorticoid receptor antagonist; NYHA, New York Heart Association.

**Table 2:** Baseline echocardiography characteristics.

	All Patients (*n* = 100)	BD (*n* = 37)	FED (*n* = 63)	*p*-Value (BD vs. FED)
LV IVS (mm)	11.0 (9.0–13.0)	11.0 (8.0–13.0)	11.0 (10.0–13.0)	**0.050**
LVEDD (mm)	52.1 ± 7.5	51.5 ± 8.6	52.5 ± 6.7	0.498
LVESD (mm)	32.5 ± 6.9	31.7 ± 6.6	33.0 ± 7.1	0.370
E/A ratio	1.7 ± 0.7	1.7 ± 0.8	1.7 ± 0.6	0.743
E/e’ ratio	14.6 ± 5.8	13.8 ± 6.5	15.1 ± 5.4	0.390
LAVi (mL/m^2^)	43.3 (32.0–58.1)	45.4 (31.2–57.9)	41.7 (32.2–58.6)	0.810
Mitral regurgitation				0.082
Grade 3	16 (16.0)	9 (24.3)	7 (11.1)	
Grade 4	84 (84.0)	28 (75.7)	56 (88.9)	
MR Rvol (mL)	70.0 (52.0–78.5)	66.0 (32.3–78.3)	72.0 (56.5–79.5)	0.342
MR EROA (mm^2^)	44.4 ± 15.9	40.4 ± 19.0	47.4 ± 12.7	0.185
MV VTI (cm)	136.3 ± 33.6	126.7 ± 44.1	141.6 ± 25.2	0.186
Prolapse volume (mL)	6.0 (1.0–9.5)	9.0 (4.5–13.5)	3.8 (0–7.1)	**0.001**
MV annulus AP diameter (mm)	35.0 ± 5.6	36.9 ± 5.8	33.7 ± 5.1	**0.017**
MV thickness (mm)	4.0 (3.0–6.0)	6.0 (5.0–7.3)	4.0 (3.0–4.5)	**<0.001**
Single leaflet prolapse	66 (66.0)	4 (10.8)	62 (98.4)	
AML (%)	8 (12.1)	0 (0)	8 (12.9)	**0.025**
PML (%)	58 (87.9)	4 (100)	54 (87.1)	**<0.001**
Bileaflet prolapse (%)	34 (34.0)	33 (89.2)	1 (1.6)	**<0.001**
Chordal rupture (%)	40 (54.1)	6 (21.4)	34 (73.9)	**<0.001**
LVEF (%)	61.0 (56.0–65.0)	60.0 (54.5–66.0)	61.0 (56.0–65.0)	0.685
LV EDVi (mL/m^2^)	85.7 ± 19.4	84.9 ± 21.5	86.1 ± 18.3	0.772
LV ESVi (mL/m^2^)	34.1 ± 10.5	34.4 ± 11.1	34.0 ± 10.1	0.863
Tricuspid regurgitation				0.174
Grade 0	3 (40.2)	19 (51.4)	20 (33.3)	
Grade 1	45 (46.2)	13 (35.1)	32 (53.3)	
Grade 2	10 (10.3)	4 (10.8)	6 (10.0)	
Grade 3	2 (2.1)	0 (0)	2 (3.3)	
Grade 4	1 (1.0)	1 (2.7)	0 (0)	
PASP (mmHg)	31.0 (26.0–42.0)	30.0 (24.5–38.0)	32.5 (27.0–43.3)	0.121
TAPSE (mm)	22.0 (18.5–25.5)	24.0 (21.0–25.8)	20.0 (16.0–27.5)	0.063

Values are mean ± standard deviation, *n* (%) or median (IQR). *p*-values ≤ 0.05 are expressed in bold. AML, anterior mitral leaflet; AP, anteroposterior; EROA, effective regurgitant orifice area; LAVi, indexed left atrial volume; LVEDD, left ventricular end-diastolic diameter; LVESD, left ventricular end-systolic diameter; LVEDVi, indexed left ventricular end-diastolic volume; LVESVi, indexed left ventricular end-systolic volume; LVEF, left ventricular ejection fraction; MR, mitral regurgitation; MV, mitral valve; PASP, pulmonary artery systolic pressure; PML, posterior mitral leaflet; Rvol, regurgitant volume; TAPSE, tricuspid annular plane systolic excursion. VTI, velocity time integral

**Table 3:** Surgical data.

	All Patients (*n* = 100)	BD (*n* = 37)	FED (*n* = 63)	*p*-Value (BD vs. FED)
Hospitalization duration (days)	10 (9–13)	10 (9–13.3)	10 (9–13)	0.582
Mitral valve repair (%)	76 (76.0)	20 (54.1)	56 (88.9)	**<0.001**
Mitral valve annuloplasty (%)	72 (94.7)	20 (100.0)	52 (92.9)	**0.002**
Neochord (%)	51 (67.1)	11 (55.0)	40 (71.4)	**0.001**
Quadrangular or triangular resection (%)	24 (31.6)	8 (40.0)	16 (28.6)	0.670
Mitral valve replacement (MVR) (%)	24 (24.0)	17 (45.9)	7 (11.1)	**<0.001**
MVR bio (%)	10 (41.7)	7 (41.2)	3 (42.9)	**0.036**
MVR mech (%)	14 (58.3)	10 (58.8)	4 (57.1)	**0.004**
Preservation of subvalvular apparatus (%)	14 (58.3)	8 (47.1)	6 (85.7)	0.090
LAA occlusion (%)	21 (21.2)	7 (19.1)	14 (22.2)	0.745

Values are *n* (%) or median (IQR). *p*-values ≤ 0.05 are expressed in bold. LAA, left atrial appendage; MR, mitral regurgitation; MVR, mitral valve replacement; PFO, patent foramen ovale; PVI, pulmonary vein isolation; TVP, tricuspid valve annuloplasty.

**Table 4:** Follow-up echocardiography characteristics.

	All Patients (*n* = 100)	BD (*n* = 37)	FED (*n* = 63)	*p*-Value (BD vs. FED)
FU since surgery (days)	278 (215–388)	278 (211–364)	288 (216–401)	0.753
LAVi (mL/m^2^)	33.2 (26.9–45.6)	33.2 (26.4–42.9)	33.3 (27.3–47.7)	0.570 °
MR grade ≥ 2 (%)	11 (11.0)	2 (5.4)	9 (14.3)	0.205
MV gradient (mmHg)				
Mean	3.0 (2.2–5.0)	3.0 (2.0–4.0)	4.0 (3.0–5.0)	0.033
Max	8.0 (6.0–12.0)	7.0 (6.0–10.0)	9.0 (6.0–12.0)	0.147
LVEF (%)	55.0 (51.0–59.5)	54.4 (48.5–56.0)	57.0 (52.3–61.0)	**<0.001 °**
LVEF < 50%	17 (17.9)	10 (27.8)	7 (11.9)	0.060 °
LV EDVi (mL/m^2^)	65.5 ± 16.2	65.8 ± 18.3	65.4 ± 14.8	0.843 °
LV ESVi (mL/m^2^)	29.8 ± 9.4	31.5 ± 11.2	28.8 ± 8.1	0.154 °
ΔLV EDVi (mL/m^2^)	−20.3 ± 17.9	−19.8 ± 19.8	−20.6 ± 16.9	0.843 °
ΔLV ESVi (mL/m^2^)	−4.4 ± 9.9	−3.0 ± 11.2	−5.3 ± 9.0	0.154 °
ΔLVEF (%)	−5.6 ± 8.3	−8.3 ± 9.6	−3.9 ± 6.9	**<0.001 °**
Relative change LVEF (%)	−8.3 ± 13.4	−12.4 ± 15.4	−5.8 ± 11.4	**0.018**
TR grade ≥ 2 (%)	2 (2.4)	1 (2.8)	1 (2.1)	1.000
PAPs (mmHg)	30.0 (25.0–32.0)	29.0 (23.8–31.0)	30.0 (26.0–34.0)	0.136 °
TAPSE (mm)	17.2 ± 4.0	16.7 ± 3.5	17.5 ± 4.4	0.891 °

Values are mean ± standard deviation, *n* (%) or median (IQR). *p*-values ≤ 0.05 are expressed in bold. ° *p*-values are corrected for respective baseline values. ΔLVEF = follow-up LVEF − baseline LVEF. Relative change in LVEF = ΔLVEF/baseline LVEF. LAVi, indexed left atrial volume; LVEDD, left ventricular end-diastolic diameter; LVESD, left ventricular end-systolic diameter; LVEDVi, indexed left ventricular end-diastolic volume; LVESVi, indexed left ventricular end-systolic volume; LVEF, left ventricular ejection fraction; MR, mitral regurgitation; MV, mitral valve; PASP, pulmonary artery systolic pressure; TAPSE, tricuspid annular plane systolic excursion; TR, tricuspid regurgitation.

**Table 5:** Univariable linear regression analysis for change in LVEF (ΔLVEF).

Variables	Univariable Analysis	
	B (95% CI)	*p*-Value
Baseline LVEF (%)	−0.74 (−0.92, −0.56)	**<0.001**
Baseline LVESVi (mL/m^2^)	0.32 (0.17, 0.47)	**<0.001**
LAVI (mL/m^2^)	−0.05 (−0.13, 0.03)	0.196
Barlow’s Disease	−4.34 (−7.71, −0.97)	**0.012**
Mitral valve replacement	−5.89 (−9.71, −2.07)	**0.003**
MR reg vol (mL)	−0.09 (−0.22, 0.04)	0.161
MR grade ≥ 2 at follow up	1.92 (−3.35, 7.20)	0.471
BMI (kg/m^2^)	0.18 (−0.26, 0.62)	0.416
Age (years)	−0.05 (−0.19, 0.09)	0.506
Atrial fibrillation	1.21 (−2.46, 4.87)	0.515
Male sex	0.986 (−2.72, 4.91)	0.599

Here, 95% CI, 95% confidence interval; BMI, body mass index; LAVi, indexed left atrial volume; LVESVi, indexed left ventricular end-systolic volume; LVEF, left ventricular ejection fraction; MR, mitral regurgitation. *p*-values ≤ 0.05 are expressed in bold.

**Table 6:** Multivariable linear regression analysis for change in LVEF (ΔLVEF).

Variables	Univariable Analysis		Collinearity Statistics	
	B (95% CI)	*p*-Value	Tolerance	VIF
Baseline LVEF (%)	−0.77 (−0.99, −0.55)	**<0.001**	0.568	1.761
Barlow’s Disease	−3.58 (−6.29, 0.88)	**0.010**	0.839	1.192
Mitral valve replacement	−2.62 (−5.76, 0.53)	0.102	0.822	1.216
LVESVi (mL/m^2^)	−0.05 (−0.20, 0.11)	0.558	0.570	1.755

Here, 95% CI, 95% confidence interval; LVESVi, indexed left ventricular end-systolic volume; LVEF, left ventricular ejection fraction; VIF, variance inflation factors. *p*-values ≤ 0.05 are expressed in bold.

## Data Availability

The data underlying this article will be shared upon reasonable request to the corresponding author.

## Supplementary Materials

The following supporting information can be downloaded at: https://www.mdpi.com/article/10.3390/jcdd11030071/s1, Table S1: Subanalysis of LV remodelling parameters at follow-up in patients who underwent mitral valve repair.
