# Supplementary material for: Mitral Valve Surgery for Mitral Regurgitation Results in Reduced Left Ventricular Ejection Fraction in Barlow’s Disease as Compared with Fibro-Elastic Deficiency
Source: J Cardiovasc Dev Dis. 2024 Feb 21;11(3):71. doi: 10.3390/jcdd11030071 (PMC10970981; doi:10.3390/jcdd11030071)
Supplement: Supplementary file 1 [file jcdd-11-00071-s001.zip › jcdd-2825273-supplementary.pdf]

Supplementary data

Table S1: Subanalysis of LV remodelling parameters at follow-up in patients who underwent mitral valve repair

	**All patients (n=76)**	**BD (n=20)**	**FED (n=56)**	**P-value (BD vs FED)**
Follow-up LV EDVi (ml/m^2)	65.1 ± 15.6	67.5 ± 17.5	64.2 ± 14.9	0.771 °
Follow-up LV ESVi (ml/m^2)	29.1 ± 9.0	30.9 ± 10.4	28.4 ± 8.3	0.603 °
Follow-up LVEF (%)	55.3 (51.8 – 60.0)	55.0 (51.0 – 56.5)	57.0 (52.2 – 61.0)	0.059 °
Δ LV EDVi (ml/m^2)	-21.5 ± 16.6	-22.8 ± 15.0	-21.0 ± 17.2	0.771 °
Δ LV ESVi (ml/m^2)	-5.8 ± 8.6	-6.1 ± 7.4	-5.7 ± 9.1	0.603 °
Δ LVEF (%)	-4.2 ± 7.5	-5.7 ± 8.5	-3.7 ± 7.1	0.059 °

Values are mean ± standard deviation or median (IQR)

° P-values are corrected for respective baseline values.

LVEDVi, indexed left ventricular end-diastolic volume; LVESVi, indexed left ventricular end-systolic volume; LVEF, left ventricular ejection fraction
